# Fic-mediated AMPylation tempers the unfolded protein response during physiological stress

**DOI:** 10.1073/pnas.2208317119

**Published:** 2022-08-01

**Authors:** Amanda K. Casey, Hillery F. Gray, Suneeta Chimalapati, Genaro Hernandez, Andrew T. Moehlman, Nathan Stewart, Hazel A. Fields, Burak Gulen, Kelly A. Servage, Karoliina Stefanius, Aubrie Blevins, Bret M. Evers, Helmut Krämer, Kim Orth

**Affiliations:** ^a^Department of Molecular Biology, University of Texas Southwestern Medical Center, Dallas, TX 75390;; ^b^HHMI, University of Texas Southwestern Medical Center, Dallas, TX 75390;; ^c^Department of Neuroscience, University of Texas Southwestern Medical Center, Dallas, TX 75390;; ^d^Department of Biochemistry, University of Texas Southwestern Medical Center, Dallas, TX 75390;; ^e^Department of Cell Biology, University of Texas Southwestern Medical Center, Dallas, TX 75390;; ^f^Department of Pathology, University of Texas Southwestern Medical Center, Dallas, TX 75390

**Keywords:** AMPylation, Fic, unfolded protein response, pancreas, ER stress

## Abstract

The proper balance of synthesis, folding, modification, and degradation of proteins, also known as protein homeostasis, is vital to cellular health and function. The unfolded protein response (UPR) is activated when the mechanisms maintaining protein homeostasis in the endoplasmic reticulum become overwhelmed. However, prolonged or strong UPR responses can result in elevated inflammation and cellular damage. Previously, we discovered that the enzyme filamentation induced by cyclic-AMP (Fic) can modulate the UPR response via posttranslational modification of binding immunoglobulin protein (BiP) by AMPylation during homeostasis and deAMPylation during stress. Loss of *fic* in *Drosophila* leads to vision defects and altered UPR activation in the fly eye. To investigate the importance of Fic-mediated AMPylation in a mammalian system, we generated a conditional null allele of *Fic* in mice and characterized the effect of *Fic* loss on the exocrine pancreas. Compared to controls, *Fic^−/−^* mice exhibit elevated serum markers for pancreatic dysfunction and display enhanced UPR signaling in the exocrine pancreas in response to physiological and pharmacological stress. In addition, both *fic^−/−^* flies and *Fic^−/−^* mice show reduced capacity to recover from damage by stress that triggers the UPR. These findings show that Fic-mediated AMPylation acts as a molecular rheostat that is required to temper the UPR response in the mammalian pancreas during physiological stress. Based on these findings, we propose that repeated physiological stress in differentiated tissues requires this rheostat for tissue resilience and continued function over the lifetime of an animal.

## Significance

During an animal’s life span, terminally differentiated cells must be resilient to fluctuating environmental and physiological stresses to assure proper function of a tissue and prevent disease. A key component of this resiliency is the maintenance of protein homeostasis. Our study reveals that the AMPylating enzyme filamentation induced by cyclic-AMP (Fic) is required in differentiated tissue for the regulation of the unfolded protein response (UPR) during both physiological and pharmacological stresses. We also propose that cells with high regenerative capacity may not require this level of regulation, as new cells will bypass the need for long-term survival. We predict that Fic is important in mitigating deleterious effects of UPR activation in a variety of tissues with UPR-associated diseases and thus holds promise as a new therapeutic target.

Protein homeostasis is regulated by proper synthesis, folding, modification, and degradation of proteins and is vital to cellular health. In the endoplasmic reticulum (ER), when the load of unfolded proteins is excessive, the unfolded protein response (UPR) is activated, triggering signaling pathways that result in changes to protein synthesis, modification, and degradation until the load of unfolded proteins is resolved. If the burden of unfolded proteins is prolonged and/or remains high, proapoptotic pathways can be activated. The activation of the UPR is, in part, regulated by the Hsp70 protein chaperone binding immunoglobulin protein (BiP), a protein that binds and helps fold proteins as they pass through the ER checkpoint and into the secretory pathway. Depending on the level of unfolded protein, complex signaling networks are activated and respond in accordance to the severity. Mild to moderate levels of UPR signaling are prominent with cell recovery and cell survival, whereas strong and prolonged UPR signaling leads to apoptosis (1, 2). These responses are mediated by three distinct ER signaling branches: inositol-requiring enzyme-1α (IRE1α); protein kinase R-like ER kinase; and activating transcription factor 6α (3).

In addition, the UPR stress can be divided into two phases: the *adaptive phase* and the *maladaptive phase* (4, 5). For the adaptive phase, the UPR induction responds to mild to moderate stress and promotes prosurvival and restorative mechanisms to promote ER homeostasis (4). By contrast, the maladaptive phase is induced by chronic and severe ER stress resulting in the activation of proinflammatory responses and apoptosis (4, 6). Disruption of ER homeostasis is predicted to play a key role in the integrated stress response and the progression of many neurodegenerative, inflammatory, and metabolic disorders (7–9). Elucidating the roles that the UPR plays in modulating ER stress provides potential therapeutic targets to treat or prevent the death of the cells subjected to prolonged ER stress and ameliorate UPR-related degenerative diseases.

Previously, the activity of BiP was shown to be regulated by a posttranslational modification (PTM) called AMPylation (10). AMPylation is a reversible PTM best described as the covalent linkage of adenosine monophosphate (AMP) to the hydroxyl group of a serine, threonine, or tyrosine residue (11). Initially discovered in the 1960s with a nucleotidyl transferase domain (12), protein AMPylation was rediscovered in 2009 with a filamentation induced by cyclic-AMP (Fic) domain from a bacterial pathogen that is also conserved in eukaryotic organisms (13, 14). To date, only two AMPylating enzymes have been identified in metazoans: Fic (also known as FicD and HYPE) localizes in the ER, and SelO localizes in the mitochondria (15, 16).

Using *Drosophila* as an animal model, we found that Fic is responsible for reversible AMPylation of BiP (10). During low ER stress or resting cells, Fic AMPylates BiP, thereby creating an inactive pool of BiP in the ER lumen (10, 17). When ER stress rises, BiP is deAMPylated and returned to an active state (10). Since this discovery, other laboratories have confirmed that this function is conserved in other metazoans, including *Caenorhabditis elegans*, rodents, and humans (16, 18, 19). We and others then demonstrated that Fic has dual catalytic activity for both the AMPylation and deAMPylation of metazoan BiP, and this activity changes depending on levels of ER stress (20, 21).

Further studies on the *Drosophila* model revealed that Fic plays a crucial role in protein homeostasis for metazoans. For *Fic* null flies (*fic^−/−^*), the gross morphology of the fly eyes appeared normal, albeit they exhibited mild vision defects. When acute physiological ER stress was induced in fly eyes by exposure to continuous light, photoreceptors in wild-type flies, but not in *fic^−/−^* mutants, could adapt (22). The damaged *fic^−/−^* eyes exhibited severe structural defects in rhabdomeres (rhodopsin-containing membranes), elevated IRE1 activity, and reduced neurotransmission (22). Flies expressing *BiP^S366A^* that were unable to be AMPylated at Ser366 phenocopied the *fic^−/−^* flies, with damaged rhabdomeres and loss of postsynaptic responses for photoreceptors stressed with continuous light. Taken together, these studies support the proposal that having a reserve of inactive AMPylated BiP that can be immediately accessed by deAMPylation allows cells to deal with physiological stress more efficiently.

Overall, we propose Fic acts as a rheostat that tempers the cellular response to stress and maintains homeostasis by deAMPylating a reserve pool of modified BiP, thereby increasing levels of active BiP to alleviate mild ER stress. When the rheostat is disrupted, either by the absence of Fic or by a mutation in BiP that hampers its AMPylation, recovery from physiologically stressed cells is hindered, as there is no resource for immediate access to additional BiP pools. In the absence of this pool, more BiP can only be provided by the time-consuming transcription and translation of de novo BiP, coincidently with the triggering of UPR.

Based on these findings, we predicted that Fic is also required for the proper regulation of physiological stress in mammals. To address this hypothesis, we generated a conditional knockout line of Fic in the mouse. As with flies, the *Fic^−/−^* animals are viable, fertile, and appear healthy upon initial inspection. However, closer characterization of *Fic^−/−^* pancreata revealed altered responses to physiological and pathological stresses, with significant changes in UPR-induced signaling. We hypothesize that without Fic, the balance and threshold between the *adaptive phase* and the *maladaptive phase* of the UPR is shifted in tissues that rely heavily on ER secretory pathway to maintain protein homeostasis. Interestingly, we observe marked resilience in wild-type flies and mice when dealing with repeated stress. By contrast, both *fic^−/−^* flies and *Fic^−/−^* mice lack the ability to efficiently recover from these stresses, resulting in damaged eyes and scarred pancreas, respectively. Taken together, our findings support the hypothesis that metazoan Fic plays a critical role by acting as a rheostat for the regulation of the UPR and protein homeostasis, likely to be important for the resilience of terminally differentiated, professional secretory cells that must respond to fluctuating needs of an organ.

## Results

### Conditional deletion of Fic.

**Fig. 1.**
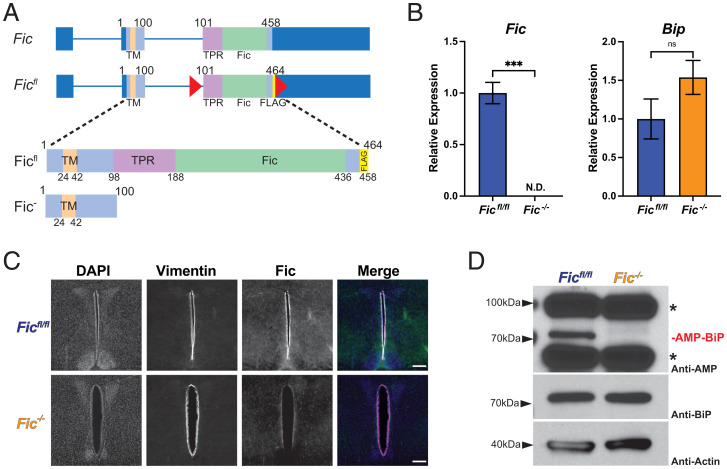
Conditional deletion of Fic. (*A*) A schematic representation of wild-type and floxed allele of Fic (*Fic^fl^*) in which LoxP sites were inserted into intron 2 and exon 3 of the Fic gene. A 6 amino acid FLAG sequence was added to the C terminus of the Fic ORF. (*B*) Quantification of *Fic* and *BiP* mRNA analyzed by qPCR from *Fic^fl/fl^* (blue bar) and *Fic^−/−^* (orange bar) mouse liver after 12 h fasting. *n* = 3. Bars indicate mean relative expression and error bars represent SE. Fic mRNA was below detection cutoff in *Fic^−/−^* samples. Statistics were performed using GraphPad Prism 9 using an unpaired *t* test. N.D., not detected; ns, not significant; ***P < 0.001. (*C*) Representative image of Fic and vimentin immunohistochemistry in coronal section of murine third ventricle. (Scale bar, 200 μM.) (*D*) Representative Western blot of liver lysates isolated from *Fic^fl/fl^* and *Fic^−/−^* mice. Blots were probed with anti-AMP, anti-BiP, and anti-actin antibodies. Asterisks (*) indicate reactivity to AMP antibody unrelated to Fic expression.

To determine the role of Fic-mediated AMPylation in the mammalian system, we chose to generate a conditional knockout line of *Fic* in the mouse. Using CRISPR-Cas9 technology, we constructed a floxed allele of *Fic* (*Fic^fl^*), in which two LoxP sites were integrated upstream of and within exon 3 (Fig. 1*A*). In addition, a single FLAG epitope was inserted into the C-terminal sequence of Fic’s coding sequence (Fig. 1*A* and *SI Appendix*, Fig. S1). Expression of Cre leads to Cre-Lox recombination that results in a nonfunctional Fic gene (Fic^−^) that is deleted for both the tetratricopeptide repeat (TPR) and Fic domains that are required for the targeting and AMPylation of BiP, respectively. The remaining truncated gene encodes only a small N-terminal peptide with the transmembrane sequence (Fig. 1*A*). *Fic^fl^* mice were bred to CAG-Cre transgenic mice (23) to generate *Fic^+/−^* mice, which were then backcrossed with *Fic^fl^* mice to generate *Fic^fl/-^* mice. Germline transmission of this allele was confirmed through PCR and sequencing (*SI Appendix*, Fig. S2). *Fic^fl/-^* mice were intercrossed to obtain sibling cohorts of *Fic^fl/fl^* and *Fic^−/−^* mice that were subsequently used in this study. *Fic^−/−^* mice were indistinguishable compared to *Fic^fl/fl^* and *Fic^fl/-^* littermates in viability, appearance, and weight (*SI Appendix*, Fig. S3).

### Fic^−/−^ mice lack Fic protein, Fic messenger RNA, and BiP AMPylation.

Using qPCR analysis of liver complementary DNA, we confirmed loss of *Fic* transcript corresponding to exon 3 of Fic, validating the Fic knockout (Fig. 1*B*). Levels of BiP transcript were not significantly altered in *Fic^fl/fl^* and *Fic^−/−^* liver. We then attempted to validate deletion of *Fic* in mice using Western blot analysis of lysates from various tissues; however, due to the low expression level of endogenous protein, we were unable to detect Fic protein in wild-type or *Fic^fl/fl^* controls (data not shown). As an alternative, we used immunofluorescence in various tissues known to express Fic and were able to detect a signal for Fic in coronal brain sections, strikingly in vimentin-positive cells lining in the third ventricle of the hypothalamus (Fig. 1*C* and *SI Appendix*, Fig. S4). In corresponding sections from *Fic^−/−^* mice, we were unable to detect a signal for Fic protein, supporting the genetic and messenger RNA expression data that Fic is not expressed in *Fic^−/−^* mice.

We next analyzed tissue for the presence or absence of Fic-mediated BiP AMPylation. Whole-cell lysates of livers from *Fic^fl/fl^* and *Fic^−/−^* littermates were analyzed for the presence of AMPylated BiP. Both Western blot analysis (Fig. 1*D*) and mass spectrometry analysis (*SI Appendix*, Fig. S5) indicate that BiP is no longer AMPylated in *Fic^−/−^* mice.

### Fasted Fic^−/−^ mice display elevated serum amylase levels.

We predicted that *Fic^−/−^* mice would have dysfunction in tissues that rely heavily on UPR to maintain proteostasis. Although many tissues are known to require this regulation, we decided to focus our initial study on Fic in the pancreas, a tissue that is well documented to rely on the UPR for proper exocrine and endocrine function (24, 25). Using the UPR fasting model, we screened *Fic^fl/fl^* and *Fic^−/−^*mice for serum markers that might indicate abnormalities in pancreatic function. A cohort of 10- to 11-week-old male mice were fasted overnight (∼14 h) with unrestricted access to water before sacrifice. Compared to *Fic^fl/fl^* and *Fic^fl/−^* controls, fic null mice have normal weights (*SI Appendix*, Fig. S3) and appear generally healthy. However, fasted serum levels of amylase were found to be significantly elevated in our *Fic^−/−^* cohorts (*SI Appendix*, Fig. S6). Of note, serum lipase levels and fasting glucose levels were not affected in our *Fic^fl/fl^* and *Fic^−/−^* mice. Since high amylase has been linked to hepatic disfunction (26), we looked at another serum marker for hepatic disfunction, aspartate aminotransferase (AST), to see if levels had changed, but levels of AST appeared normal in both *Fic^fl/fl^* and *Fic^−/−^* mice (*SI Appendix*, Fig. S6). Histopathological analysis of both the pancreas and liver revealed no detectable defect in either tissue (*SI Appendix*, Fig. S7).

### Physiological stressed fasted-fed Fic^−/−^ mice display altered UPR signaling in the pancreas.

**Fig. 2.**
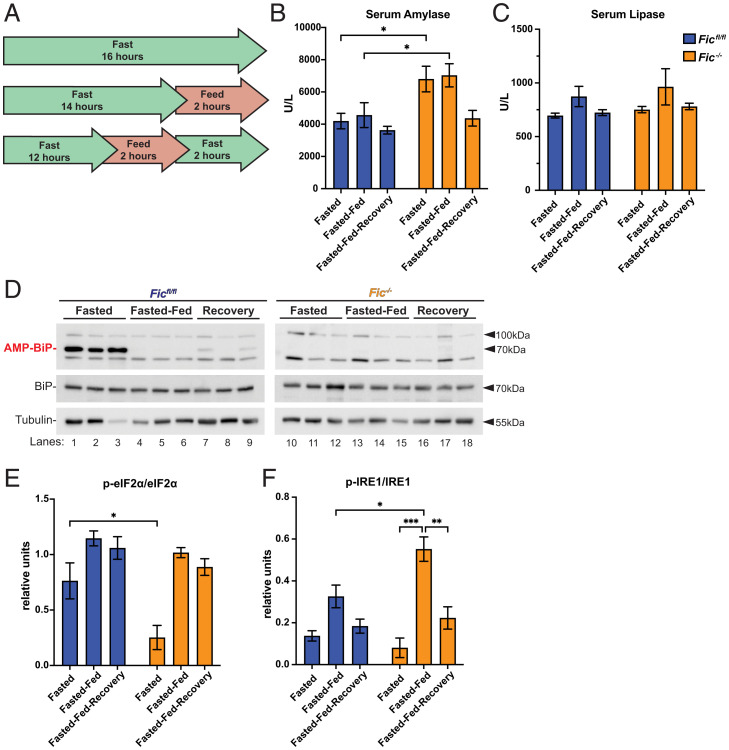
Response of fasted-fed Fic^fl/fl^ and Fic^−/−^ pancreata. (*A*) A schematic representation of fasted, fasted-fed, and fasted-fed-recovery experimental conditions. (*B*) Quantification of serum amylase in *Fic^fl/fl^ and Fic^−/−^* mice under fasted, fasted-fed, and fasted-fed-recovery conditions. Statistics were performed using GraphPad Prism 9 using a two-way ANOVA. *n* = 8. Bars indicate mean and error bars represent SE. (*C*) Quantification of serum lipase in *Fic^fl/fl^ and Fic^−/−^* mice under fasted, fasted-fed, and fasted-fed-recovery conditions. *n* = 8. Bars indicate mean and error bars represent SE. (*D*) Representative Western blot of P2 lysate fractions isolated from *Fic^fl/fl^* and *Fic^−/−^* pancreas. Blots were probed with anti-AMP, anti-BiP, and anti-tubulin antibodies. (*E*) Quantification of relative anti-phospho-eiF2α to anti-p-eiF2α shown in *SI Appendix*, Fig. S8. Statistics were performed using GraphPad Prism 9 using two-way ANOVA. *n* = 3. (*F*) Quantification of relative phospho-Ire1 shown in *SI Appendix*, Fig. S9. Statistics were performed using GraphPad Prism 9 using a two-way ANOVA. *n* = 3. *P < 0.05; **P < 0.01; ***P < 0.001.

Based on the observed changes in serum amylase in our *Fic^−/−^* mice, we predicted that *Fic^−/−^* mice would show changes in UPR activation in the exocrine pancreatic tissue. To determine if this was the case, we utilized a mild physiological stress of fasting-feeding to activate the UPR in the exocrine pancreas. Fasting-feeding is a well-established method used to activate mild UPR in the pancreas through the stimulation of pancreatic digestive enzyme synthesis (27, 28). In this study, sibling cohorts of 6- to 8-week-old male mice were split into three groups: fasted (for 16 h), fasted-fed (fasted 14 h, fed 2 h), and fasted-fed-recovery (fasted 12 h, fed 2 h, fasted 2 h) (Fig. 2*A*).

Compared to *Fic^fl/fl^* controls, elevated serum amylase levels, but not lipase, were detected in fasted and fasted-fed groups of *Fic^−/−^* mice (Fig. 2 *B* and *C*). Notably, serum amylase levels were not increased in *Fic^−/−^* mice in the fasted-fed-recovery group. Western blot analysis of pancreatic lysates in *Fic^fl/fl^* samples from a separate cohort (6- to 8-week-old female mice) confirmed that BiP AMPylation is present during fasted conditions and lost during fasted-fed conditions. Of note, AMPylation of BiP returns in the fasted-fed-recovery group, albeit reduced (Fig. 2*D*). Western analysis of phospo-eIF2α and eIF2α showed reduced eIF2α phosphorylation in fasted *Fic^−/−^* mice, whereas phospho-eIF2α was not found to differ during fasted-fed and fasted-fed-recovery conditions (Fig. 2*E* and *SI Appendix*, Fig. S8). Western blot analysis performed on Phos-tag gels indicated that activation of IRE1 was observed to be stronger in fasted-fed *Fic^−/−^* mice compared to *Fic^fl/fl^* controls (Fig. 2*F* and *SI Appendix*, Fig. S9).

**Fig. 3.**
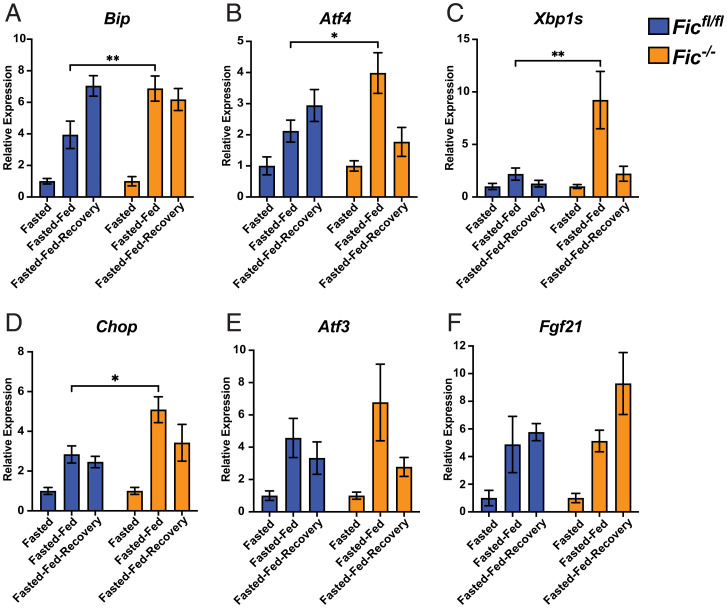
UPR signaling in fasted-fed Fic^fl/fl^ and Fic^−/−^ pancreata. (*A*–*F*) Quantification of *BiP, ATF4, Xbp1s, Chop, ATF3,* and *Fgf21* mRNA analyzed by qPCR from *Fic^fl/fl^* (blue bar) and *Fic^−/−^* (orange bar) mouse pancreas after fasting, fast-feeding, and fast-feed-recovery. Expression values were normalized to that of the housekeeping gene *U36B4.* Bars indicate mean relative expression compared to fasted controls, and error bars represent SE. Statistics were performed using GraphPad Prism 9 using a two-way ANOVA. *n* = 8. *P < 0.05; **P < 0.01.

To further assess the activation of the UPR in the pancreas, levels of UPR-induced transcripts were analyzed using qPCR. As previously reported (28), feeding after fasting resulted in a significant increase in UPR-regulated transcripts in mice (Fig. 3 and *SI Appendix*, Fig. S10). In *Fic^fl/fl^* mice, levels of *Fic* were found to increase during fasted-fed conditions and then diminish during fasted-fed-recovery (*SI Appendix*, Fig. S10). Notably, in *Fic^−/−^* mice, many UPR-induced transcripts were elevated compared to *Fic^fl/fl^* controls. Levels of *BiP, Atf4, Xbp1s,* and *Chop* were all found to be significantly increased in *Fic^−/−^* mice during fasted-fed conditions (Fig. 3 *A*–*D*).

However, not all UPR-responsive genes followed this pattern. Changes in levels of *Atf3* and *Fgf21* were not found to differ significantly between *Fic^fl/fl^* and *Fic^−/−^* mice upon fast-feeding (Fig. 3 *E* and *F*). Of note, no significant differences of these transcript levels were observed in the fasted-fed-recovery group, signifying that this difference in UPR induction is short lived.

### Stressing Fic^−/−^ mice with pancreatic caerulein treatment reveals changes in UPR response.

**Fig. 4.**
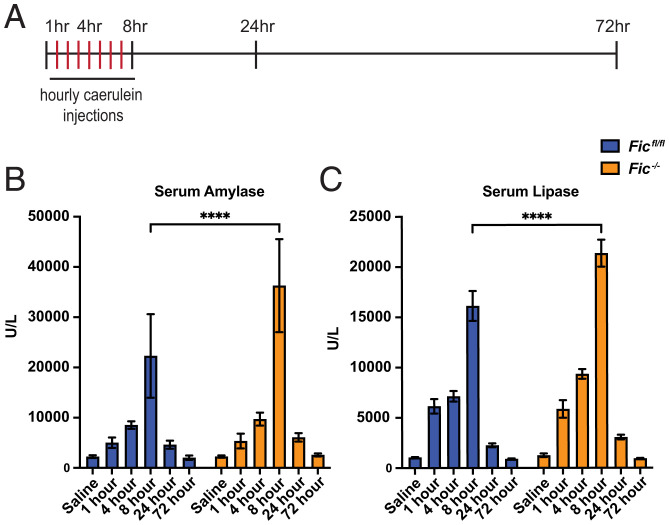
Caerulein-induced pancreatitis in Fic^fl/fl^ and Fic^−/−^ mice. (*A*) A schematic representation of caerulein-induced acute pancreatitis induction. (*B*, *C*) Quantification of serum amylase and lipase in *Fic^fl/fl^ and Fic^−/−^* mice over 72 h of caerulein-induced acute pancreatitis. Bars indicate mean and error bars represent SE. Statistics were performed using GraphPad Prism 9 using an two-way ANOVA. *n* = 5–7. ****P < 0.0001.

Next, we wanted to assess the fitness of *Fic^−/−^* mice in response to a maladaptive, pathological ER stress and injury to the pancreas. For this, we used a well-established model for pancreatic injury by injecting mice with caerulein, a cholecystokinin analog and secretagogue (29, 30). In this study, pancreatitis was induced in 8- to 10-week-old female cohorts with seven hourly intraperitoneal (IP) injections of caerulein or, as a control, saline. Animals were sacrificed at 1, 4, 8, 24, and 72 h after the first injection (Fig. 4*A*). Immediately upon sacrifice, serum was collected from each mouse, and the pancreas was collected for RNA and histopathology.

As previously reported (29), treatment with caerulein induces acute pancreatitis. Analysis of serum revealed elevated levels of amylase and lipase in caerulein-treated mice. In addition, levels of serum amylase and lipase were significantly increased in *Fic^−/−^* mice compared to *Fic^fl/fl^* at 8 h after the first injection of caerulein (Fig. 4 *B* and *C*).

To assess if caerulein injury differentially alters the UPR response in *Fic^−/−^* mice compared to *Fic^fl/fl^* mice, we assessed the levels of UPR-induced transcripts in saline and caerulein-treated mice by qPCR. (*SI Appendix*, Fig. S11). As previously reported, UPR-induced transcripts are elevated upon caerulein-induced injury and resolve over the course of injury recovery (31–34). Whereas both *Fic^fl/fl^* and *Fic^−/−^* mice displayed similar levels of increased UPR transcript induction, the peak of transcript changes appeared earlier in *Fic^−/−^* mice.

One hour after the first injection of caerulein, levels of the UPR-induced transcripts *Ask1*, *Egr1*, and *Fgf21* were found to be elevated in *Fic^−/−^* mice compared to wild-type mice (*SI Appendix*, Fig. S11 *A–C*). However, at 4 h after the first injection, *Ask1* and *Egr1* levels were comparable in wild-type and *Fic^−/−^* mice, and *Fgf21* transcript levels were significantly lower than in wild type. Similarly, at 4 h after the first injection, levels of *Atf3*, *Atf4*, and *Hmox1* transcripts were elevated in *Fic^−/−^* mice compared to *Fic^fl/fl^* (*SI Appendix*, Fig. S11 *D–F*). By 8 h after the first injection, transcript levels of *Atf3* and *Atf4* were comparable between *Fic^fl/fl^* and *Fic^−/−^* mice, whereas *Hmox1* transcript levels were significantly lower in *Fic^−/−^* mice. *BiP* transcript levels also dropped significantly in *Fic^−/−^* mice at 8 h (*SI Appendix*, Fig. S11*G*). Expression patterns of *Chop* were also significantly altered, with reduced transcript levels for *Fic^−/−^* both at 4 and 8 h after the first caerulein injection (*SI Appendix*, Fig. S11*H*). Levels of *Xbp1s* appeared unaffected in *Fic^−/−^* mice (*SI Appendix*, Fig. S11*I*). Of note, the changes in *Fic* transcript levels in caerulein-treated mice mirrored the changes seen in *Xbp1s* transcript levels (*SI Appendix*, Fig. S11*J*). With the exception of *Chop* and *Xbp1s*, UPR transcripts appeared to peak and diminish in the pancreas of caerulein-treated *Fic^−/−^* mice early compared to *Fic^fl/fl^*.

### Fic is required for recovery of ER stress in both *Drosophila* and mice.

Next, we asked if Fic was required for cellular recovery after prolonged ER stress. In previous studies using *Drosophila* as a model, we found that *fic^−/−^* flies were maladaptive to light stress. In this system, 3 d of constant light caused damage to ommatidial structures found in the *fic^−/−^* compound eye and enhanced UPR activation (22). In these previous experiments, we found that the defects in rhabdomere integrity were completely reversible in wild-type flies, but, significantly, *fic^−/−^* flies only partially recovered after 3 d of normal light/dark conditions.

**Fig. 5.**
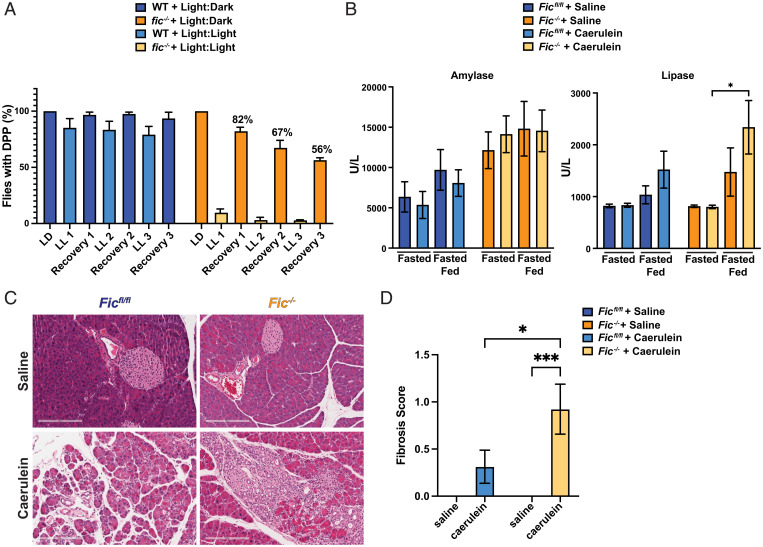
Fic is required for recovery after stress. (*A*) Quantification of flies possessing intact deep DPPs at each indicated time. Bars indicate mean, and error bars represent SE. N = ∼150. (*B*) Quantification of serum amylase and lipase in *Fic^fl/fl^ and Fic^−/−^* mice 7 d after caerulein-induced acute pancreatitis. Bars indicate mean, and error bars represent SE. Statistics were performed using GraphPad Prism 9 using an two-way ANOVA. *n* = 6–8. (*C*) Representative hematoxylin and eosin stained images of pancreas of *Fic^fl/fl^* and *Fic^−/−^* mice 7 d after treatment with saline or caerulein. (*D*) Quantification of pancreas with fibrosis, *Fic^fl/fl^* and *Fic^−/−^* mice 7 d after treatment with saline or caerulein. Bars indicate mean, and error bars represent SE. Statistics were performed using GraphPad Prism 9 using an 2-way ANOVA. *n* = 13–16. *P < 0.05; ***P < 0.001.

As an extension of these previous experiments, we wanted to ask whether a repetitive version of this stress in flies results in a degenerative effect in affected tissues. To test this, we repeated treatments of constant light (LL) for 3 d, followed by 3 d of recovery (LD) on the same cohort of flies and scored for presence or lack of intact deep pseudopupils (DPPs), a visual indicator for disruption of ommatidial structure (Fig. 5*A*). As previously observed, for the first round of LL and LD treatment, wild-type flies completely recovered but *fic^−/−^* flies showed only an 82% recovery. We found that with a second round of LL and LD treatment, wild-type flies retained their ability to fully recover but *fic^−/−^* flies had only a 67% recovery. Finally, by the third repeat of this stress, wild-type flies continued to fully recover, whereas only 56% of *fic^−/−^* flies retained intact DPPs.

Based on the changes in UPR response kinetics and increased serum amylase and lipase levels in caerulein-treated *Fic^−/−^* mice, we hypothesized that *Fic^−/−^* mice might also exhibit more severe and prolonged histopathological changes in the pancreas upon caerulein-induced pancreatitis. The severity of pancreatitis in each saline- and caerulein-treated mouse was scored by severity of pancreatic edema, inflammatory infiltrate, and necrosis, as previously described (35). Based on these criteria, *Fic^fl/fl^* and *Fic^−/−^* mice exhibited comparable gross histological changes to the pancreas, with inflammation, edema, and necrosis observed in both cohorts over the course of the 72 h experiment (*SI Appendix*, Figs. S12 and S13 and Table S1), revealing no detectable difference in pancreatitis severity tissue after one round of acute caerluein injury.

Previous reports indicate wild-type mice typically fully recover from caerulein-induced acute pancreatitis by 7 d postinjury (36). We wanted to assess how *Fic^fl/fl^* and *Fic^−/−^* mice recovered from caerulein-induced pancreatitis. A cohort of 6- to 8-week-old *Fic^fl/fl^* and *Fic^−/−^* male mice was induced for pancreatitis with seven hourly IP injections of saline or caerulein and left to recover for 7 d. Mice were then split into two groups: a fasted and fasted-fed group as previously described (Fig. 2*A*). Immediately upon sacrifice, RNA and tissue for histopathology from the pancreas of each mouse were collected.

As previously described (Fig. 2*B*), *Fic^−/−^* mice had elevated serum amylase levels upon fasting and fasting-feeding, but the elevation of serum amylase was no longer affected, indicating that both *Fic^fl/fl^* and *Fic^−/−^* mice had recovered from the initial caerulein-induced injury (Fig. 5*B*). Notably, serum lipase of caerulein-treated *Fic^−/−^* mice after fast-feeding was elevated. Moreover, histopathological scoring of the pancreas showed an increased incidence of fibrosis in caerulein-treated *Fic^−/−^* mice than *Fic^fl/fl^* after 7 d of recovery (Fig. 5 *C* and *D* and Table 1). These data suggest that *Fic^−/−^* mice have increased scarring and reduced capacity for recovery from tissue damage.

**Table 1.** Histopathological scoring of caerulein pancreatitis after 1 wk recovery

Genotype	Treatment	Edema	Inflammation	Necrosis	Fibrosis	Total
*Fic^fl/fl^*	Saline	0 ± 0	0 ± 0	0 ± 0	0 ± 0	0 ± 0
*Fic^fl/fl^*	Caerulein	0 ± 0	0.13 ± 0.085	0 ± 0	0.31 ± 0.18	0.44 ± 0.24
*Fic^−/−^*	Saline	0 ± 0	0 ± 0	0 ± 0	0 ± 0	0 ± 0
*Fic^−/−^*	Caerulein	0.08 ± 0.077	0.39 ± 0.18	0.077 ± 0.077	0.92 ± 0.25	1.46 ± 0.48

Average scores +/− SEM.

## Discussion

The UPR is crucial for the maintenance of protein homeostasis during physiological stress of cells with high secretory capacity. When cellular stress levels reach maladaptive levels, recovery from stress becomes challenging due to prolonged attenuated protein synthesis of nonstress-responsive genes and activation of apoptotic machinery. Thus, regulation of the UPR must be tightly coordinated to meet the needs of the tissue. To understand the role of reversible AMPylation of BiP in protein homeostasis in a mammalian system, we generated a conditional knockout of Fic in mice. We speculated that tissues reliant on secretion might be most affected by the deletion of Fic and therefore focused our initial efforts on the pancreas. Using both a fast-feeding and a caerulein-induced pancreatic injury model, we observed changes to UPR signaling and physiology of the pancreas suggestive of exocrine pancreas disfunction in *Fic^−/−^* mice. Analysis of UPR markers for these experiments revealed changes in the timing and duration of the UPR transcriptional response (Fig. 3 and *SI Appendix*, Fig. S11).

Furthermore, analysis of tissue recovery after light-induced or caerulein-induced damage in *Drosophila* eyes and mouse pancreas, respectively, indicates that loss of Fic reduces recovery from ER stress-associated tissue damage in both animal models (Fig. 5). Therefore, the loss of Fic-mediated AMPylated BiP leaves tissues vulnerable to irreversible damage with chronic and repeated stresses.

Our and other groups have proposed that inactivating PTMs on BiP could provide a mechanism by which rapidly changing physiological fluctuations of the ER stress can be nimbly regulated (37). AMPylation of BiP by Fic allows for a pool of inactive chaperone to remain in the ER without deleterious consequences to protein folding that might otherwise be hindered in the presence of excess chaperone (37, 38). Previous studies indicate that the pool of inactivated BiP is significant in various cell types, ∼40% in fasted pancreas and over 50% in unstressed *Drosophila* S2 cells (10, 37). This inactive pool of BiP can then be readily activated to address increasing loads of unfolded proteins in cells with rapidly fluctuating demands on protein synthesis and secretion, such as the pancreas, while tempering the activation of the UPR.

**Fig. 6.**
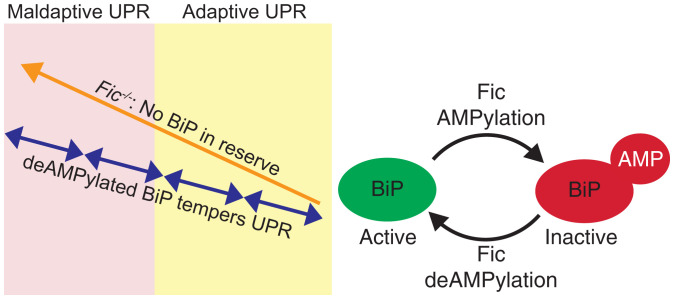
Model of Fic tempering of UPR.

We predict that Fic provides a necessary level of regulation of the UPR to properly adjust protein homeostasis in tissue with frequent physiological ER stress (Fig. 6). By keeping a readily accessible pool of inactive BiP, cells can provide a nimble response to ER stress through a short burst of UPR activation. Rapid deAMPylation of BiP results in additional active chaperone much faster than what can be accomplished by new protein synthesis. This results in smaller, more moderate pulses of UPR signaling under repeated physiological stresses, keeping the cell in the beneficial *adaptive phase* of the UPR. As Fic is a UPR-responsive gene, it is possible that as these stresses are repeated, a larger pool of inactive BiP may be generated over time, resulting in a more robust rapid response to unfolded proteins in the ER in these adapted tissues.

Cells without Fic regulation of BiP lack this pool of chaperone on standby, resulting in prolonged and elevated UPR signaling, as a delayed response requires more chaperone via transcription and translation to accommodate the increased physiological stress. This is supported by our qPCR analysis of UPR-responsive genes in *Fic^−/−^* mice under both physiological and pharmacological stresses (Fig. 3 and *SI Appendix*, Fig. S11). Repetitive stress in *Fic^−/−^* tissues would result in amplified UPR, leading to progression into the maladaptive phase of the UPR and tissue damage over the lifetime of the tissue. Our data point to evidence of this in the exocrine pancreas, where elevated UPR signaling and serum amylase indicate functional disruption. As elevated serum amylase is one of the first key clinical indicators of pancreas dysfunction, we suspect additional and continued physiological stresses to *Fic^−/−^* tissue will lead to increased prevalence of disease.

The pancreas primarily comprises terminally differentiated professional secretory cells with limited regenerative capability. Therefore, the pancreas must employ mechanisms to ensure resilience to repetitive stress in order to last and properly function for the lifetime of the animal. Herein, we provide evidence that Fic provides one such mechanism through the moderation of the UPR during physiological stress. Similarly, a wild-type fly eye has the capacity to recover from the physiological stress of continuous light. In the absence of the Fic rheostat, the *fic^−/−^* eyes are challenged over time and lose the potential to regenerate rhabdomere integrity. Analogously, we observe more scarring in the injured *Fic^−/−^* pancreas.

Many studies to date have used tissue culture cell lines as a model to study the UPR in which a chemical stress is applied to cells resulting in a very strong, and frequently irreversible, induction of the UPR (39). Under these conditions, tissue culture cells respond in basically two ways, cell death or replication, allowing for new cells to overcome the stress. These options are far from optimal for differentiated cells within a tissue where cellular function needs to be maintained for survival of the organ and/or animal. We predict that many subtleties of UPR regulation will only be apparent under such physiological stresses in the context of specific tissues. Thus, it is not surprising that studies with tissue culture models have only exhibited very subtle differences in activation of UPR in the absence of Fic (19). Systems in an animal that use cells with high regenerative capacity and shorter life spans may not require Fic mediation of the UPR, as turnover and replenishment with new cells will bypass the need of rheostat. This is consistent with observations by other groups with a Fic deletion model (40). In sum, we predict that terminally differentiated postmitotic cells will be principally reliant upon the Fic-mediated rheostat to maintain a healthy response to continuing physiological stress over an animal’s lifetime.

Whereas this study focuses on this one tissue only, we speculate that other tissues with professional secretory, terminally differentiated cells that must adapt to fluctuating stress will be similarly affected in the *Fic^−/−^* mouse. We propose the presence of Fic rheostat allows for tempering of the UPR response by maintaining a window for reversible UPR response that is critical for maintenance of protein homeostasis. The importance of this window has been highlighted with the treatment of UPR stress with the pharmacological agent ISRIB (integrated stress response inhibitor) where it is only observed to be efficacious during the adaptive phase of UPR (9). Future studies with ISRIB and *Fic^−/−^* mice will be useful for understanding the importance of the Fic-mediated rheostat and treatment of disease.

For the health of an animal, it is critical to maintain resilience in terminally differentiated cells during repeated physiological stress to prevent disease. We predict that Fic regulation of the UPR will play a role in mitigating the deleterious effects of UPR activation in a variety of tissues with UPR-associated diseases, including retinal degeneration, atherosclerosis, metabolic syndrome, and various neurodegenerative disorders. Our future studies will focus on the identification of tissues in which Fic plays a role in the regulation of the UPR and the physiological consequences of the absence of Fic-mediated regulation of the UPR.

## Materials and Methods

Detailed descriptions of the experimental methods are provided in *SI Appendix*, *SI Materials and Methods*. These include mouse protocols, generation of conditional Fic knockout, histology, quantitative real-time PCR, immunofluorescence of brain sections, mass spectrometry analysis, Western blot analysis, fly stocks and rearing conditions, and deep pseudopupil analysis.

## Supplementary Material

Supplementary File

## Data Availability

All study data are included in the article and/or supporting information.

## References

[1] B. M. Gardner, D. Pincus, K. Gotthardt, C. M. Gallagher, P. Walter, Endoplasmic reticulum stress sensing in the unfolded protein response. Cold Spring Harb. Perspect. Biol. 5, a013169 (2013). 23388626 10.1101/cshperspect.a013169 PMC3578356

[2] A. Bertolotti, Y. Zhang, L. M. Hendershot, H. P. Harding, D. Ron, Dynamic interaction of BiP and ER stress transducers in the unfolded-protein response. Nat. Cell Biol. 2, 326–332 (2000). 10854322 10.1038/35014014

[3] P. Walter, D. Ron, The unfolded protein response: From stress pathway to homeostatic regulation. Science 334, 1081–1086 (2011). 22116877 10.1126/science.1209038

[4] K. Meyerovich, F. Ortis, F. Allagnat, A. K. Cardozo, Endoplasmic reticulum stress and the unfolded protein response in pancreatic islet inflammation. J. Mol. Endocrinol. 57, R1–R17 (2016). 27067637 10.1530/JME-15-0306

[5] C. Hetz, K. Zhang, R. J. Kaufman, Mechanisms, regulation and functions of the unfolded protein response. Nat. Rev. Mol. Cell Biol. 21, 421–438 (2020). 32457508 10.1038/s41580-020-0250-z PMC8867924

[6] C. J. Adams, M. C. Kopp, N. Larburu, P. R. Nowak, M. M. U. Ali, Structure and molecular mechanism of ER stress signaling by the unfolded protein response signal activator IRE1. Front. Mol. Biosci. 6, 11 (2019). 30931312 10.3389/fmolb.2019.00011 PMC6423427

[7] J. Lee, U. Ozcan, Unfolded protein response signaling and metabolic diseases. J. Biol. Chem. 289, 1203–1211 (2014). 24324257 10.1074/jbc.R113.534743 PMC3894306

[8] M. K. Brown, N. Naidoo, The endoplasmic reticulum stress response in aging and age-related diseases. Front. Physiol. 3, 263 (2012). 22934019 10.3389/fphys.2012.00263 PMC3429039

[9] M. Costa-Mattioli, P. Walter, The integrated stress response: From mechanism to disease. Science 368, eaat5314 (2020). 32327570 10.1126/science.aat5314 PMC8997189

[10] H. Ham , Unfolded protein response-regulated Drosophila Fic (dFic) protein reversibly AMPylates BiP chaperone during endoplasmic reticulum homeostasis. J. Biol. Chem. 289, 36059–36069 (2014). 25395623 10.1074/jbc.M114.612515 PMC4276871

[11] A. K. Casey, K. Orth, Enzymes involved in AMPylation and deAMPylation. Chem. Rev. 118, 1199–1215 (2017). 28819965 10.1021/acs.chemrev.7b00145 PMC5896785

[12] H. S. Kingdon, B. M. Shapiro, E. R. Stadtman, Regulation of glutamine synthetase. 8. ATP: Glutamine synthetase adenylyltransferase, an enzyme that catalyzes alterations in the regulatory properties of glutamine synthetase. Proc. Natl. Acad. Sci. U.S.A. 58, 1703–1710 (1967). 4867671 10.1073/pnas.58.4.1703 PMC223983

[13] M. L. Yarbrough , AMPylation of Rho GTPases by Vibrio VopS disrupts effector binding and downstream signaling. Science 323, 269–272 (2009). 19039103 10.1126/science.1166382

[14] B. Gulen, A. Itzen, Revisiting AMPylation through the lens of Fic enzymes. Trends Microbiol. 30, 350–363 (2022). 34531089 10.1016/j.tim.2021.08.003

[15] A. Sreelatha , Protein AMPylation by an evolutionarily conserved pseudokinase. Cell 175, 809–821.e19 (2018). 30270044 10.1016/j.cell.2018.08.046 PMC6524645

[16] C. A. Worby , The fic domain: Regulation of cell signaling by adenylylation. Mol. Cell 34, 93–103 (2009). 19362538 10.1016/j.molcel.2009.03.008 PMC2820730

[17] S. Preissler , AMPylation targets the rate-limiting step of BiP’s ATPase cycle for its functional inactivation. eLife 6, e29428 (2017). 29064368 10.7554/eLife.29428 PMC5667935

[18] M. C. Truttmann , The *Caenorhabditis elegans* protein FIC-1 is an AMPylase that covalently modifies heat-shock 70 family proteins, translation elongation factors and histones. PLoS Genet. 12, e1006023 (2016). 27138431 10.1371/journal.pgen.1006023 PMC4854385

[19] S. Preissler , AMPylation matches BiP activity to client protein load in the endoplasmic reticulum. eLife 4, e12621 (2015). 26673894 10.7554/eLife.12621 PMC4739761

[20] A. K. Casey , Fic-mediated deAMPylation is not dependent on homodimerization and rescues toxic AMPylation in flies. J. Biol. Chem. 292, 21193–21204 (2017). 29089387 10.1074/jbc.M117.799296 PMC5743091

[21] S. Preissler, C. Rato, L. Perera, V. Saudek, D. Ron, FICD acts bifunctionally to AMPylate and de-AMPylate the endoplasmic reticulum chaperone BiP. Nat. Struct. Mol. Biol. 24, 23–29 (2017). 27918543 10.1038/nsmb.3337 PMC5221731

[22] A. T. Moehlman, A. K. Casey, K. Servage, K. Orth, H. Krämer, Adaptation to constant light requires Fic-mediated AMPylation of BiP to protect against reversible photoreceptor degeneration. eLife 7, e38752 (2018). 30015618 10.7554/eLife.38752 PMC6066327

[23] K. Sakai, Ji. Miyazaki, A transgenic mouse line that retains Cre recombinase activity in mature oocytes irrespective of the Cre transgene transmission. Biochem. Biophys. Res. Commun. 237, 318–324 (1997). 9268708 10.1006/bbrc.1997.7111

[24] A. H. Lee, G. C. Chu, N. N. Iwakoshi, L. H. Glimcher, XBP-1 is required for biogenesis of cellular secretory machinery of exocrine glands. EMBO J. 24, 4368–4380 (2005). 16362047 10.1038/sj.emboj.7600903 PMC1356340

[25] S. J. Pandol, F. S. Gorelick, A. Lugea, Environmental and genetic stressors and the unfolded protein response in exocrine pancreatic function—A hypothesis. Front. Physiol. 2, 8 (2011). 21483727 10.3389/fphys.2011.00008 PMC3070477

[26] G. A. Coté, J. H. Gottstein, A. Daud, A. T. Blei; Acute Liver Failure Study Group, The role of etiology in the hyperamylasemia of acute liver failure. Am. J. Gastroenterol. 104, 592–597 (2009). 19223884 10.1038/ajg.2008.84 PMC3641762

[27] M. D. Sans, S. H. Lee, L. G. Alecy, J. A. Williams, Feeding activates protein synthesis in mouse pancreas at the translational level without increase in mRNA. Am. J. Physiol. Gastrointest. Liver Physiol. 287, G667–G675 (2004). 15117679 10.1152/ajpgi.00505.2003

[28] L. Yang , A Phos-tag-based approach reveals the extent of physiological endoplasmic reticulum stress. PLoS One 5, e11621 (2010). 20661282 10.1371/journal.pone.0011621 PMC2905412

[29] J. J. Hyun, H. S. Lee, Experimental models of pancreatitis. Clin. Endosc. 47, 212–216 (2014). 24944983 10.5946/ce.2014.47.3.212 PMC4058537

[30] M. D. Sans, J. A. Williams, Translational control of protein synthesis in pancreatic acinar cells. Int. J. Gastrointest. Cancer 31, 107–115 (2002). 12622421 10.1385/IJGC:31:1-3:107

[31] A. S. Kowalik , Mice lacking the transcription factor Mist1 exhibit an altered stress response and increased sensitivity to caerulein-induced pancreatitis. Am. J. Physiol. Gastrointest. Liver Physiol. 292, G1123–G1132 (2007). 17170023 10.1152/ajpgi.00512.2006

[32] K. C. Coate , FGF21 is an exocrine pancreas secretagogue. Cell Metab. 25, 472–480 (2017). 28089565 10.1016/j.cmet.2016.12.004 PMC5299054

[33] G. Hernandez , Pancreatitis is an FGF21-deficient state that is corrected by replacement therapy. Sci. Transl. Med. 12, eaay5186 (2020). 31915301 10.1126/scitranslmed.aay5186 PMC7034981

[34] L. Zhou , P53 activated by ER stress aggravates caerulein-induced acute pancreatitis progression by inducing acinar cell apoptosis. Dig. Dis. Sci. 65, 3211–3222 (2020). 31974911 10.1007/s10620-020-06052-5

[35] S. Wildi , Suppression of transforming growth factor beta signalling aborts caerulein induced pancreatitis and eliminates restricted stimulation at high caerulein concentrations. Gut 56, 685–692 (2007). 17135311 10.1136/gut.2006.105833 PMC1942167

[36] A. Lugea , Pancreas recovery following cerulein-induced pancreatitis is impaired in plasminogen-deficient mice. Gastroenterology 131, 885–899 (2006). 16952557 10.1053/j.gastro.2006.06.023 PMC1636452

[37] J. E. Chambers, K. Petrova, G. Tomba, M. Vendruscolo, D. Ron, ADP ribosylation adapts an ER chaperone response to short-term fluctuations in unfolded protein load. J. Cell Biol. 198, 371–385 (2012). 22869598 10.1083/jcb.201202005 PMC3413365

[38] J. H. Otero, B. Lizák, L. M. Hendershot, Life and death of a BiP substrate. Semin. Cell Dev. Biol. 21, 472–478 (2010). 20026282 10.1016/j.semcdb.2009.12.008 PMC2883687

[39] C. M. Oslowski, F. Urano, Measuring ER stress and the unfolded protein response using mammalian tissue culture system. Methods Enzymol. 490, 71–92 (2011). 21266244 10.1016/B978-0-12-385114-7.00004-0 PMC3701721

[40] N. McCaul , Deletion of mFICD AMPylase alters cytokine secretion and affects visual short-term learning in vivo. J. Biol. Chem. 297, 100991 (2021). 34419450 10.1016/j.jbc.2021.100991 PMC8441161

