# Supplementary material for: Fic-mediated AMPylation tempers the unfolded protein response during physiological stress
Source: Proc Natl Acad Sci U S A. 2022 Aug 1;119(32):e2208317119. doi: 10.1073/pnas.2208317119 (PMC9371680; doi:10.1073/pnas.2208317119)
Supplement: Supplementary File [file pnas.2208317119.sapp.pdf]

PNAS

Supplementary Information for

Fic-mediated AMPylation tempers the unfolded protein response during physiological stress.

Amanda K. Casey[1], Hillery F. Gray[1,2], Suneeta Chimalapati[1,2], Genaro Hernandez[1], Andrew T. Moehlman[3], Nathan Stewart[1,2], Hazel A. Fields[1], Burak Gulen[1], Kelly A. Servage[1,2], Karoliina Stefanius[1,2,5], Aubrie Blevins[1], Bret M. Evers[6], Helmut Krämer[3,5], Kim Orth[1,2, 4]

*Corresponding author: Kim Orth[1]
Email: kim.orth@utsouthwestern.edu

This PDF file includes:

Figures S1 to S13
Tables S1 to S2

Supplementary Information Text

SI Materials and Methods

Mice

The Institutional Animal Care and Use Committee of the University of Texas Southwestern Medical Center approved all experiments. Mice were housed in a specific pathogen-free facility and fed a standard chow diet (#2016 Harlan Teklad). C57BL/6J mice were purchased through the University of Texas Southwestern Mouse Breeding Core. For fasting studies, mice were separated into three groups fasted (for 16 hours), fasted-fed (fasted 14-hours, fed 2 hours), and fasted-fed-recovery (fasted 12 hours, fed 2 hours, fasted 2 hours). All mice were singly housed and fasted overnight (~12-14 hours) with unrestricted access to water. After fasting, the fasted-fed group were provided unrestricted access to food for a 2 hour window and immediately sacrificed. The fasted-fed-recovery group provided unrestricted access to food for a 2 hour window and then food was removed for an additional 2 hours before sacrifice. Immediately upon sacrifice, blood was collected from each mouse and the pancreas was collected for analysis. Acute pancreatitis was induced as previously described (32) by administration of seven hourly intraperitoneal injections of caerulein (50 µg/kg) (Tocris Bioscience). Mice in the control group were injected with saline. For serum analysis, blood was collected in Microvette serum collection tubes (Sarstedt) and centrifuged at 10,000xg at 4°C for 15 minutes. Serum analysis was performed by *UT Southwestern Metabolic Phenotyping Core* using VITROS MicroSlide™ technology

Generation of conditional Fic knockout

Fic$^{fl/fl}$ mice (C57Bl/6N background) harboring the conditional floxed Fic alleles were generated using CRISPR/Cas9 reagents at the Transgenic Technology Center of UT Southwestern Medical Center. The guide RNAs and donor ssODNs were designed to insert one *loxP* site upstream and another *loxP* site downstream of exon 3. The sgRNA sequences are 5' ggggacctcccaatgtagag 3' (upstream) and 5' gctggcggttagggcctcac 3' (downstream). Guides were selected using the CRISPR Design Tool (http://tools.genome-engineering.org). The crRNA and tracRNA were annealed and mixed with Cas9 protein to form a ribonucleotide protein complex. The donor ssODNs (IDT, Inc.) was added to the mixture and the cocktail was microinjected into the cytoplasm of fertilized, pronuclear staged eggs isolated from superovulated females. The eggs were incubated in media containing cytochalasin-B immediately before and during microinjection to improve egg survival. Alternatively, CRISPR reagents were delivered to the cytoplasm via electroporation using either a Nepa21 Super Electroporator (NEPAGENE, Ichikawa, Japan) or Gene Pulser (BioRad, Hercules, CA, USA). The surviving eggs were transferred into the oviducts of day 0.5 pseudopregnant recipient ICR females (Envigo, Inc.) to produce putative founder mice. Founder mice were identified via PCR using the primer set 5' cagtgcagccatactgtagg 3' and 5' ccatgtggcttctgtgactc 3' for the upstream *loxP* and primer set 5' gccctggcccattacaaac 3' and 5' gctatcccctgccactcag 3' for the downstream *loxP* site and the amplicons were submitted for Sanger sequencing. F0 mice were bred with C57Bl/6N or J mice to obtain F1 mice heterozygous for the floxed Fic allele (*Fic*$^{+/fl}$), which were then intercrossed to produce *Fic*$^{fl/fl}$ mice. For deletion of *Fic*, *Fic*$^{fl/fl}$ mice were bred with C57Bl/6N mice carrying a *CAG-Cre* transgene(23). To confirm that *Fic* was deleted, 20 ng genomic DNA extracted from tail snips was used for genotyping by PCR with the following primers; upstream (5' gggggtggttcaaggaag 3' and downstream 5' ctgcaacctcctactggc 3' followed by restriction digestion with EcoR1 and BamH1).

Histology

Mouse pancreas and liver were harvested and fixed in 10% neutral buffered formalin overnight at 4°C. Paraffin sections were embedded by the UT Southwestern (UTSW) Molecular Pathology Core. Samples were sectioned, and hematoxylin and eosin (H&E)–stained. H&E-stained pancreas was

graded as previously described on an equal weight score (from 0 to 3) for edema (0 = absent; 1 = focally increased between lobules; 2 = diffusely increased; 3 = acini disrupted and separated), inflammatory infiltration (0 = absent; 1 = in ducts, around ductal margins; 2 = in the parenchyma, <50% of the lobules; 3 = in the parenchyma, >50% of the lobules), necrosis (0 = absent; 1 = periductal necrosis, <5% of cells; 2 = focal necrosis, 5 to 20% of cells; 3 = diffuse parenchymal necrosis, 20 to 50% of cells), and total severity score (sum of edema, inflammatory infiltrate, and necrosis scores). In addition H&E-stained pancreas from mice 1 week after caerulein treatment were graded for fibrosis (0 = absent; 1 = mild fibrosis between acini within at least one lobule, 2 = moderate fibrosis between acini with acinar drop-out in at least one lobule, 3 = severe fibrosis between acini with acinar drop-out in at least one lobule)

Quantitative real-time PCR
RNA from pancreas and liver was extracted using RNA Stat-60 (Iso-Tex Diagnostics). Complementary DNA (cDNA) was generated from RNA (2 µg) using the High-Capacity cDNA Reverse Transcription Kit (Life Technologies). qPCR was performed by the SYBR Green method (41). Primer sequences for the genes analyzed can be found in **Table S2**. *U36B4* (NM_007475) was used as the reference mRNA. Experiments were performed on a BioRad CFX Touch and analyzed with CFX Maestro Software.

Immunofluorescence of Brain Sections
Mouse brains were harvested and fixed in 10% neutral buffered formalin overnight at 4°C. Brains were equilibrated in 30% sucrose solution in 1x PBS and *embedded* with optimal cutting temperature (OCT) compound and frozen on dry ice. Samples were sectioned via cryostat at a thickness of 50µM. Sections were incubated for 1 hour in blocking buffer (5% normal serum, 1% BSA, 0.3% Triton X-100 in PBS), followed by overnight incubation at 4°C with primary antibodies anti-vimentin (Abcam) and anti-HYPE (custom antibody from Thermo/Fischer against GDVRPFIRFIAKCTET peptide) in blocking buffer. Sections were washed in PBS three times, followed by incubation for 1 hour with Alexa Fluor–conjugated secondary antibodies against the primary antibody's host species in blocking buffer. Sections were washed in PBS three times, stained with 4′,6-diamidino-2-phenylindole (DAPI) (Life Technologies), and mounted with Aqua-Poly/Mount (Polysciences). Images were acquired with a Zeiss Axioscan.Z1 slide scanner provided by the Whole Brain Microscopy Facility at UTSW.

Mass Spectrometry Analysis
Endogenous BiP samples from $Fic^{-/-}$ and $Fic^{fl/fl}$ mouse livers were run on SDS-PAGE gel and stained with Coomassie blue dye prior to analysis by mass spectrometry. Gel bands containing proteins were reduced with DTT for 1hr at 56°C and alkylated with iodoacetamide for 45min at room temperature in the dark. Samples were then digested with trypsin (MS grade) overnight at 37°C. Tryptic peptides were de-salted via solid phase extraction (SPE) prior to LC-MS/MS analysis. Experiments were performed on a Thermo Scientific EASY-nLC liquid chromatography system coupled to a Thermo Scientific Orbitrap Fusion Lumos mass spectrometer. To generate MS/MS spectra, MS1 spectra were first acquired in the Orbitrap mass analyzer (resolution 120,000). Peptide precursor ions were isolated and fragmented using high-energy collision-induced dissociation (HCD). The resulting MS/MS fragmentation spectra were acquired in the ion trap. MS/MS spectral data was searched using the search engine Mascot (Matrix Science). Precursor and product ion mass tolerances were set to 15 ppm and 0.6 Da, respectively. Three missed cleavages were allowed. Modifications included carbamidomethylation of cysteine (+57.021 Da), oxidation of methionine (+15.995 Da), and AMPylation of serine/threonine/tyrosine (+329.053 Da). MS/MS spectra of AMPylated peptides were manually searched and verified. Biological replicates of each sample were analyzed.

Western Blot Analysis

Pancreas was washed in PBS and homogenized in isolation buffer (20mM Tris pH 7.4, 5mM NaF, 1mM EDTA, 1mM EGTA, 250mM Sucrose, PMSF, PhosSTOP (Roche), Protease Inhibitor Cocktail (Roche)) with a Teflon-coated dounce homogenizer. Lysates were centrifuged at 600xg for 10 minutes three times at 4°C to remove nuclei and cellular debris (P1). The supernatant (S1) was collected and centrifuged at 13,000xg for 15 minutes to pellet ER and mitochondrial membranes (P2) for western blot analysis. Supernatant (S2) was reserved for western blot analysis. Lysates were separated by SDS-PAGE and transferred to PVDF membranes. Phos tag analysis was performed as previously described (28).Blots were probed with anti-AMP 17g6 (gift from Aymelt Itzen), anti-GRP78 (Abcam), anti-tubulin (Abcam), anti-eif2A (Cell Signaling), anti-EIF2S1-phospho (Abcam), anti-IRE1 (Cell Signaling), and anti-IRE1-phospho (Novis). Membranes were then incubated with horseradish peroxidase–conjugated secondary antibodies (Cell Signaling Technology) against the primary antibody's host species for 1 hour. Membranes were developed using the ECL substrate solution (Bio-Rad). Quantification of western blots was performed using NIH ImageJ software. Band densities were measured and subtracted from background.

Fly stocks and rearing conditions

Bloomington Stock Center provided w^{1118} (BS# 3605). The fic^{30C} flies was previously described (20). All flies were reared on standard molasses fly food, under room temperature conditions. For light treatments, flies were collected within one to two days of enclosing, and placed in 5cm diameter vials containing normal food, with no more than 25 flies, and placed at either LD (lights ON 8am/lights OFF 8pm) or LL. The same intensity white LED light source was used for both conditions and flies were kept the same distance away from the light source, which amounted to approximately 500 lux. LD and LL treatments were done at 25°C.

Deep pseudopupil analysis

Flies were anesthetized on CO_2 and aligned with one eye facing up. Using a stereoscopic dissection microscope, each fly was scored for presence or loss of the deep pseudopupil (42), and the percentage of flies with intact pseudopupils was calculated. For each genotype/treatment, over 50 flies were scored per replica and three biological replicas were performed (n=3).

```
FicD_Flag      MILMPMASVVAVAEPKWVSVWGRFLWMALLSMALGSLLALLLPLGVVEEHCLAVLRGFHL      60
FicD_WT        MILMPMASVVAVAEPKWVSVWGRFLWMALLSMALGSLLALLLPLGVVEEHCLAVLRGFHL      60
FicD_Null      MILMPMASVVAVAEPKWVSVWGRFLWMALLSMALGSLLALLLPLGVVEEHCLAVLRGFHL      60
               ************************************************************

FicD_Flag      LRSKLDRAQPVVPKCTSLCTELSVSSRDAGLLTVKTTASPAGKLEAKAALNQALEMKRQG      120
FicD_WT        LRSKLDRAQPVVPKCTSLCTELSVSSRDAGLLTVKTTASPAGKLEAKAALNQALEMKRQG      120
FicD_Null      LRSKLDRAQPVVPKCTSLCTELSVSSRDAGLLTVKTTASP--------------------      100
               ****************************************

FicD_Flag      KRGKAHKLFLHALKMDPGFVDALNEFGIFSEEDKDIIQADYLYTRALTISPFHEKALVNR      180
FicD_WT        KRGKAHKLFLHALKMDPGFVDALNEFGIFSEEDKDIIQADYLYTRALTISPFHEKALVNR      180
FicD_Null      ------------------------------------------------------------      100

FicD_Flag      DRTLPLVEEIDQRYFSVIDSKVKKVMSIPKGSSALRRVMEETYYHHIYHTVAIEGNTLTL      240
FicD_WT        DRTLPLVEEIDQRYFSVIDSKVKKVMSIPKGSSALRRVMEETYYHHIYHTVAIEGNTLTL      240
FicD_Null      ------------------------------------------------------------      100

FicD_Flag      SEIRHILETRYAVPGKSLEEQNEVIGMHAAMKYINTTLVSRIGSVTMDDMLEIHRRVLGY      300
FicD_WT        SEIRHILETRYAVPGKSLEEQNEVIGMHAAMKYINTTLVSRIGSVTMDDMLEIHRRVLGY      300
FicD_Null      ------------------------------------------------------------      100

FicD_Flag      VDPVEAGRFRRTQVLVGHHIPPHPRDVEKQMQEFTQWLNSEDAMNLHPVEFAALAHYKLV      360
FicD_WT        VDPVEAGRFRRTQVLVGHHIPPHPRDVEKQMQEFTQWLNSEDAMNLHPVEFAALAHYKLV      360
FicD_Null      ------------------------------------------------------------      100

FicD_Flag      YIHPFIDGNGRTSRLLMNLILMQAGYPPITIRKEQRSEYYHVLEVANEGDVRPFIRFIAK      420
FicD_WT        YIHPFIDGNGRTSRLLMNLILMQAGYPPITIRKEQRSEYYHVLEVANEGDVRPFIRFIAK      420
FicD_Null      ------------------------------------------------------------      100

FicD_Flag      CTEVTLDTLLLATTEYSVALPEAQPNHSGFKETLPVDYKDHDGF*    464
FicD_WT        CTEVTLDTLLLATTEYSVALPEAQPNHSGFKETLPVRP*------    458
FicD_Null      ---------------------------------------------    100
```

Fig. S1. Multiple sequence alignment of Fic. The predicted protein sequences of *Fic*^{fl} (FicD_FLAG), *Fic* (FicD_WT), and *Fic*$^{-}$ (FicD_Null) were aligned. Fic^{fl} sequence is identical to Fic with the addition of a 6 amino acid FLAG sequence on the C-terminus of the protein. Fic^{-} sequence results in a truncated 100 amino acid protein.

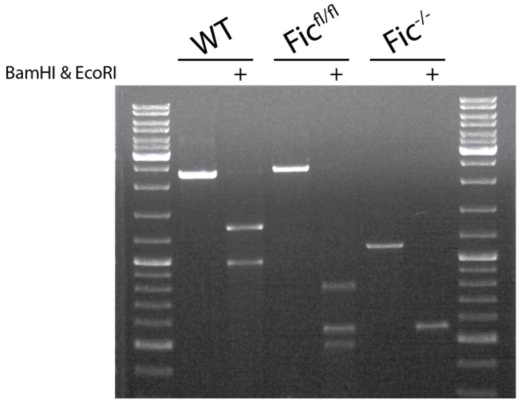

Fig. S2. PCR genotyping of Fic alleles. Representative agarose gel of PCR amplicons and digests. Primers were used that amply across the modified region of Fic, producing a 2306bp Fic^{+} amplicon, 2407bp Fic^{fl} amplicon, and 1118bp Fic^{-} amplicon. Digestion of Fic amplicons with BamHI and EcoRI result in DNA fragments of 1318 and 988 bp (Fic^{+}), 802, 565, 556, and 484 (Fic^{fl}), and 556 and 562 (Fic^{-}).

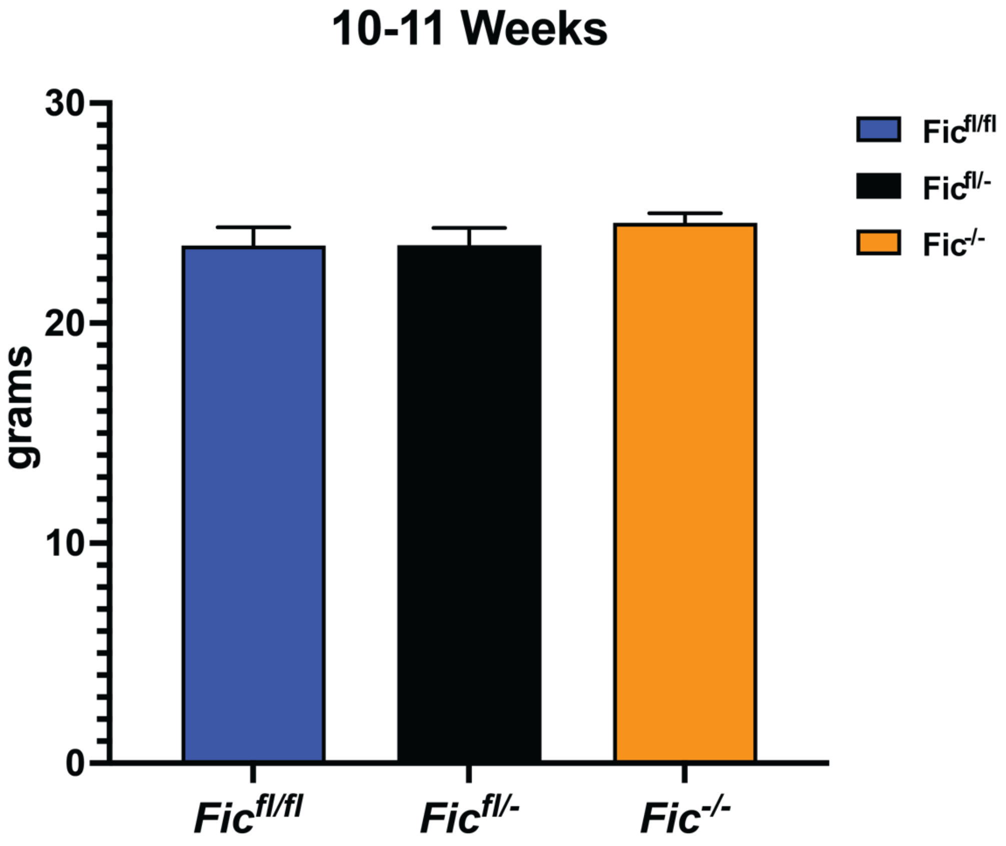

Fig. S3. 10-11 week old male *Fic*$^{fl/fl}$ (N= 8), *Fic*$^{fl/-}$ (N=9), and *Fic*$^{-/-}$ (N=9) littermates were weighed before fasting. Graph represent weight of mice. Bars indicate mean weight and error bars represent standard error.

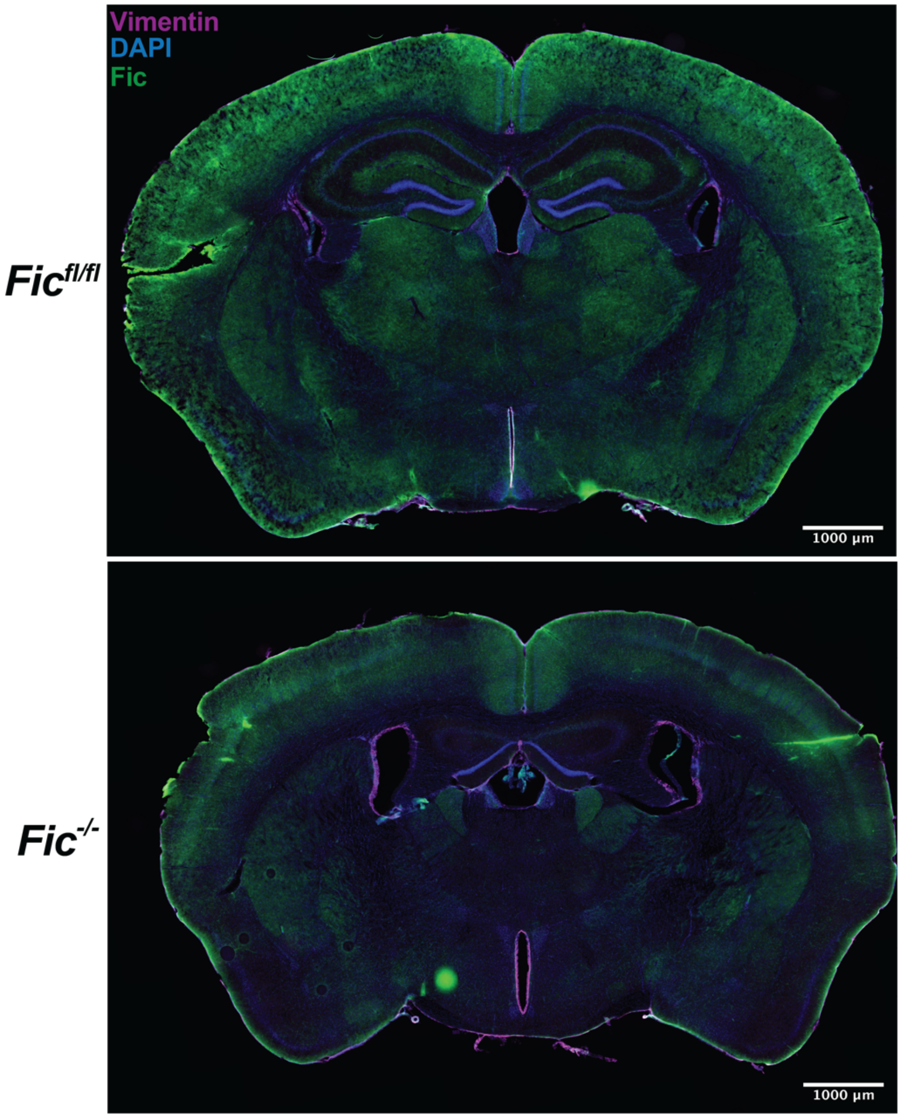

Fig. S4. Representative image of anti-Fic and anti-Vimentin immunohistochemistry in coronal section of murine brain. Scale bar, 1000μM.

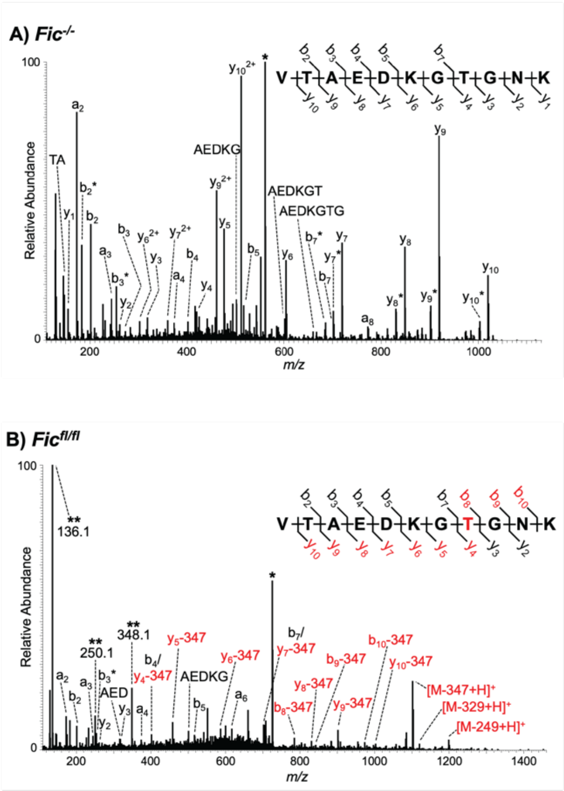

Fig. S5. MS/MS spectra of **A)** unmodified peptide ion VTAEDKGTGNK from Fic$^{-/-}$ mouse and **B)** AMPylated peptide ion VTAEDKGTGNK from Fic$^{fl/fl}$ mouse. The AMPylated threonine residue is highlighted in red while AMPylation was not observed in Fic$^{-/-}$. The precursor $[M+2H]^{2+}$ ion is labeled with a single asterisk (*) and spectra were generated via HCD fragmentation. Fragment ions containing the AMPylated threonine residue (red) show characteristic mass shifts corresponding to loss of the AMP group (-347 Da). Unique ions corresponding to neutral loss of the AMP group (labeled with **) are also present at 136.1, 250.1, and 348.1 Da. B- and y- fragment ions labeled with a single asterisk (*) correspond to neutral loss of H_2O (-18 Da).

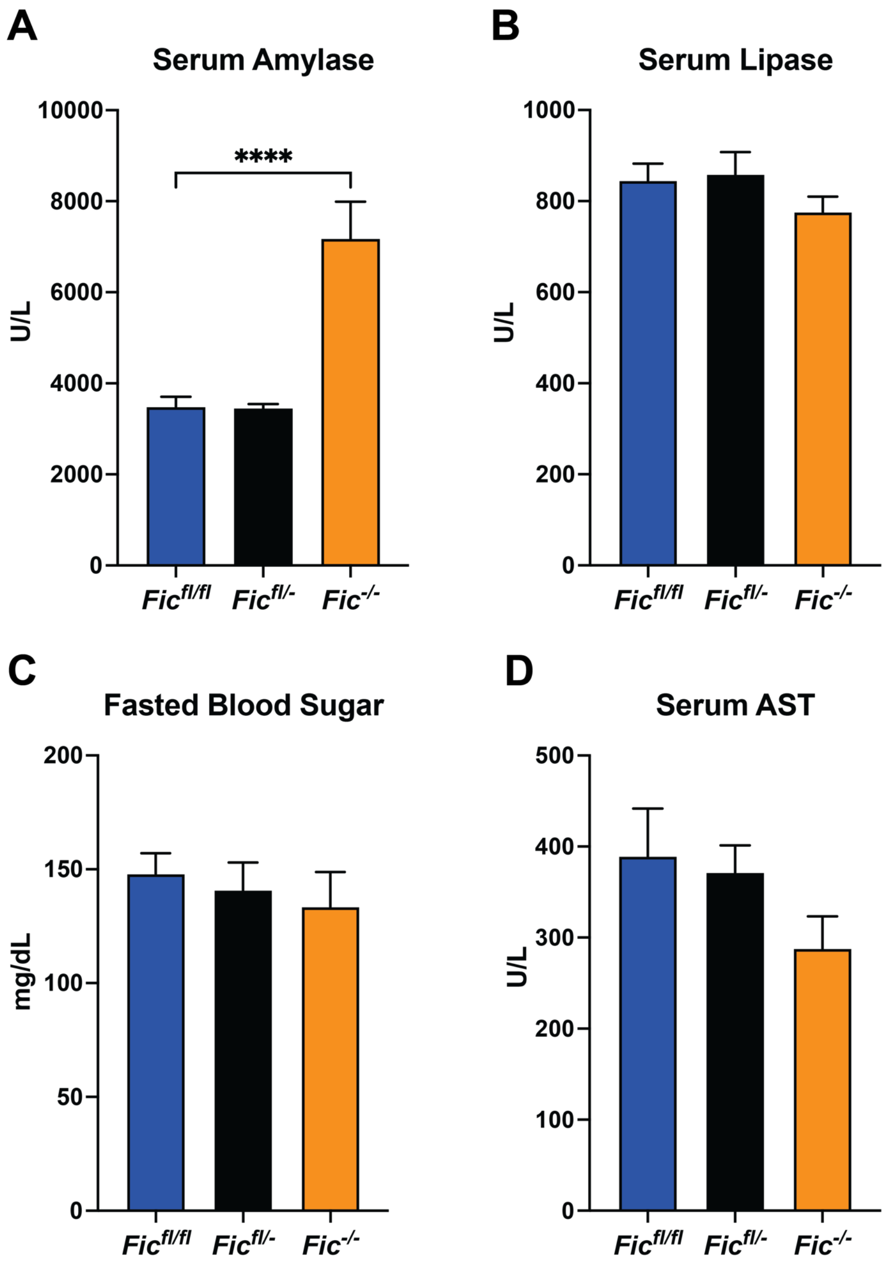

Fig. S6. A) Quantification of serum Amylase in $Fic^{fl/fl}$, $Fic^{fl/-}$, *and* $Fic^{-/-}$ mice under fasted conditions. B) Quantification of serum Amylase in $Fic^{fl/fl}$ *and* $Fic^{-/-}$ mice under fasted conditions. C) Quantification of blood glucose in $Fic^{fl/fl}$ *and* $Fic^{-/-}$ mice under fasted conditions. D) Quantification of serum aspartate aminotransferase (AST) in $Fic^{fl/fl}$ *and* $Fic^{-/-}$ mice under fasted conditions. Bars indicate mean and error bars represent standard error. Statistics were performed using GraphPad Prism 9 using an 1-way ANOVA. N=8-9. ****, $p < 0.0001$.

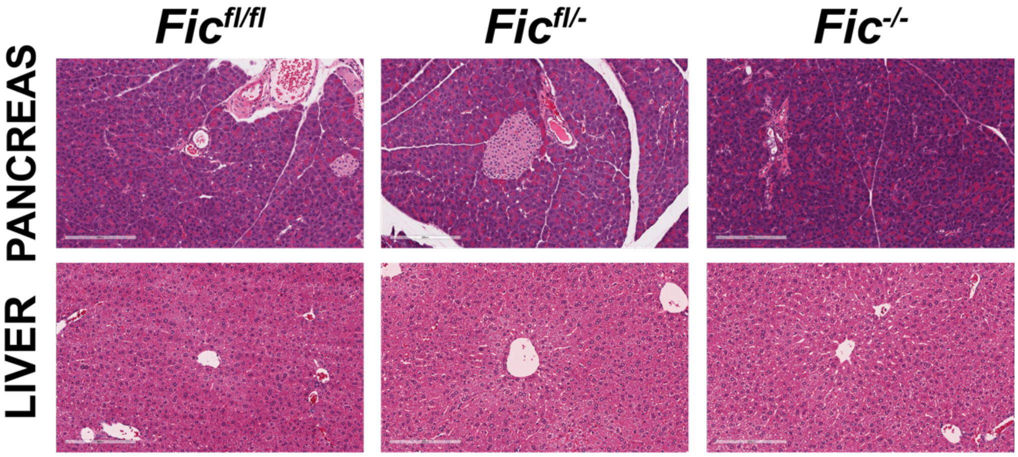

Fig. S7. Representative hematoxylin and eosin stained images of pancreas and liver from $Fic^{fl/fl}$, $Fic^{fl/-}$, *and* $Fic^{-/-}$ mice. Scale bar, 200μM

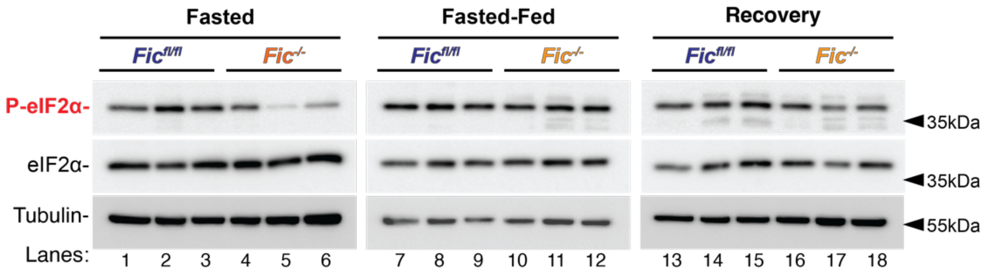

Fig. S8. Representative western blot of S2 lysate fractions from isolated from *Fic*$^{fl/fl}$ and *Fic*$^{-/-}$ pancreas. Blots were probed with anti-phospho-eiF2α, anti-eiF2α, and anti-Tubulin antibodies.

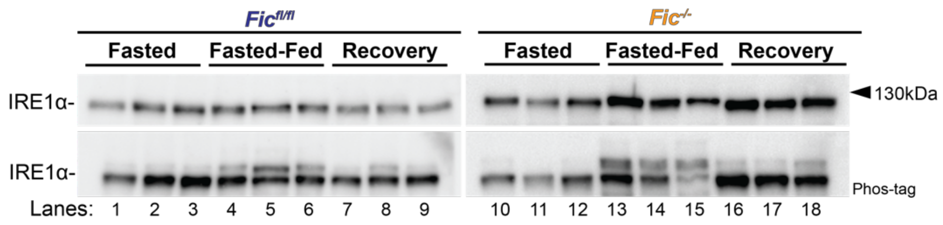

Fig. S9. Representative western blot of P2 lysate fractions from isolated from *Fic*$^{fl/fl}$ and *Fic*$^{-/-}$ pancreas. Blots were probed with anti-Ire1 antibodies.

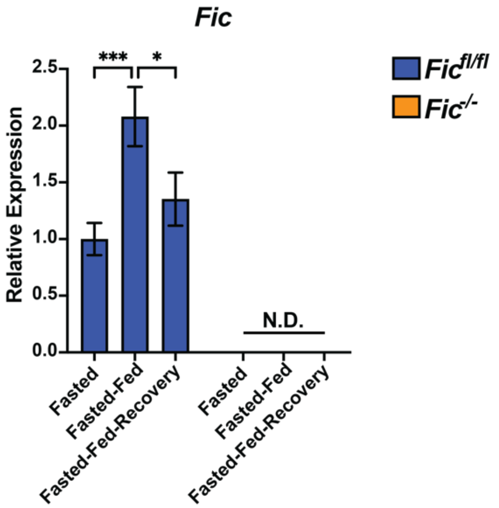

Fig. S10. Quantification of *Fic* mRNA analyzed by qPCR from *Fic*$^{fl/fl}$ (blue bar) and *Fic*$^{-/-}$ (orange bar) mouse pancreas after fasting, fast-feeding, and fast-feed-recovery. Expression values were normalized to that of the housekeeping gene *U36B4.* Bars indicate mean relative expression compared to fasted controls, and error bars represent standard error. Fic mRNA was below detection cutoff in *Fic*$^{-/-}$ samples. Statistics were performed using GraphPad Prism 9 using an 2-way ANOVA. N=8. N.D., not detected; *, $p < 0.05$; ***, $p < 0.001$.

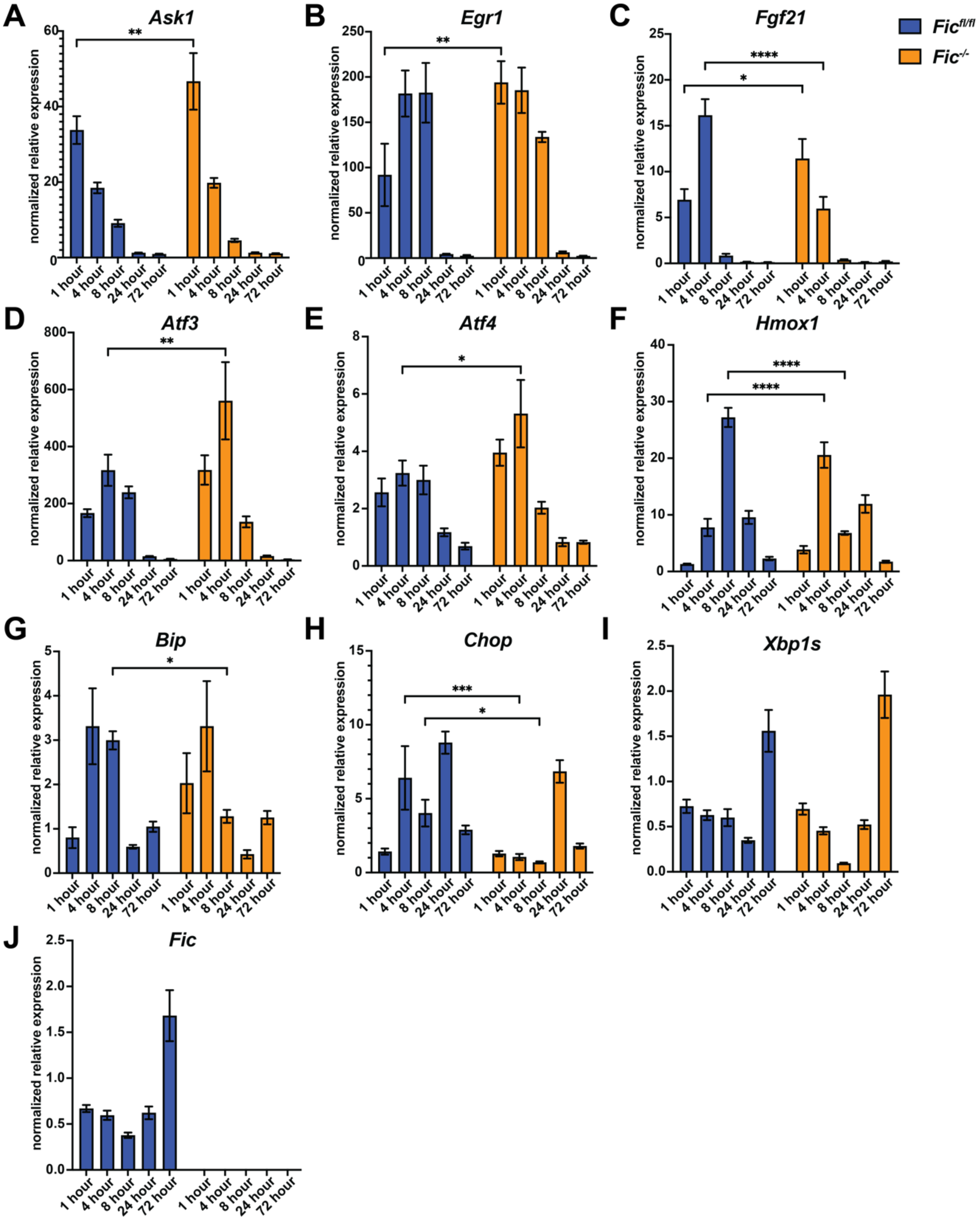

Figure S11. ***UPR signaling in caerulein-induced acute pancreatitis in Fic$^{fl/fl}$ and Fic$^{-/-}$ mice.*** A-J) Quantification of *Ask1*, *Egr1*, *Fgf21*, *Atf3*, *Atf4*, *Hmox1*, *BiP*, *Chop, Xbp1s,* and *Fic* mRNA analyzed by qPCR from *Fic$^{fl/fl}$* (blue bar) and *Fic$^{-/-}$* (orange bar) mouse pancreas during 72 hours of caerulein-induced acute pancreatitis and recovery. Expression values were normalized to that of the housekeeping gene *U36B4.* Bars indicate mean relative expression compared to Saline-treated controls at each timepoint. Error bars represent standard error. Statistics were performed using GraphPad Prism 9 using an 2-way ANOVA. N=5-7. *, $p < 0.05$; **, $p < 0.01$; ***, $p < 0.001$; ****, $p < 0.0001$.

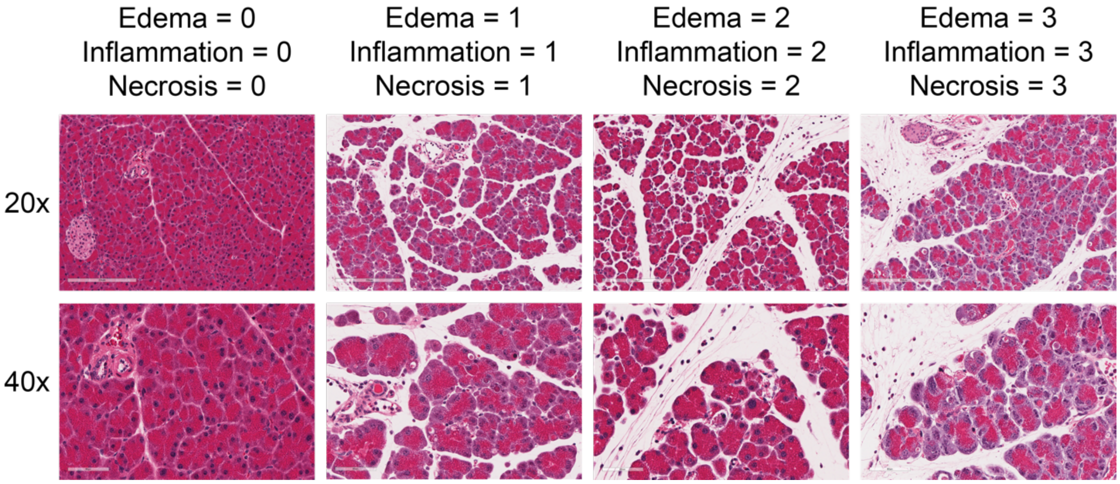

Fig. S12. Representative hematoxylin and eosin stained images of pancreas with severity of pancreatic edema, inflammatory infiltrate, necrosis of 0, 1, 2 and 3. Images at 20x (scale bar, 200μM) and 40x (scale bar, 60μM) shown.

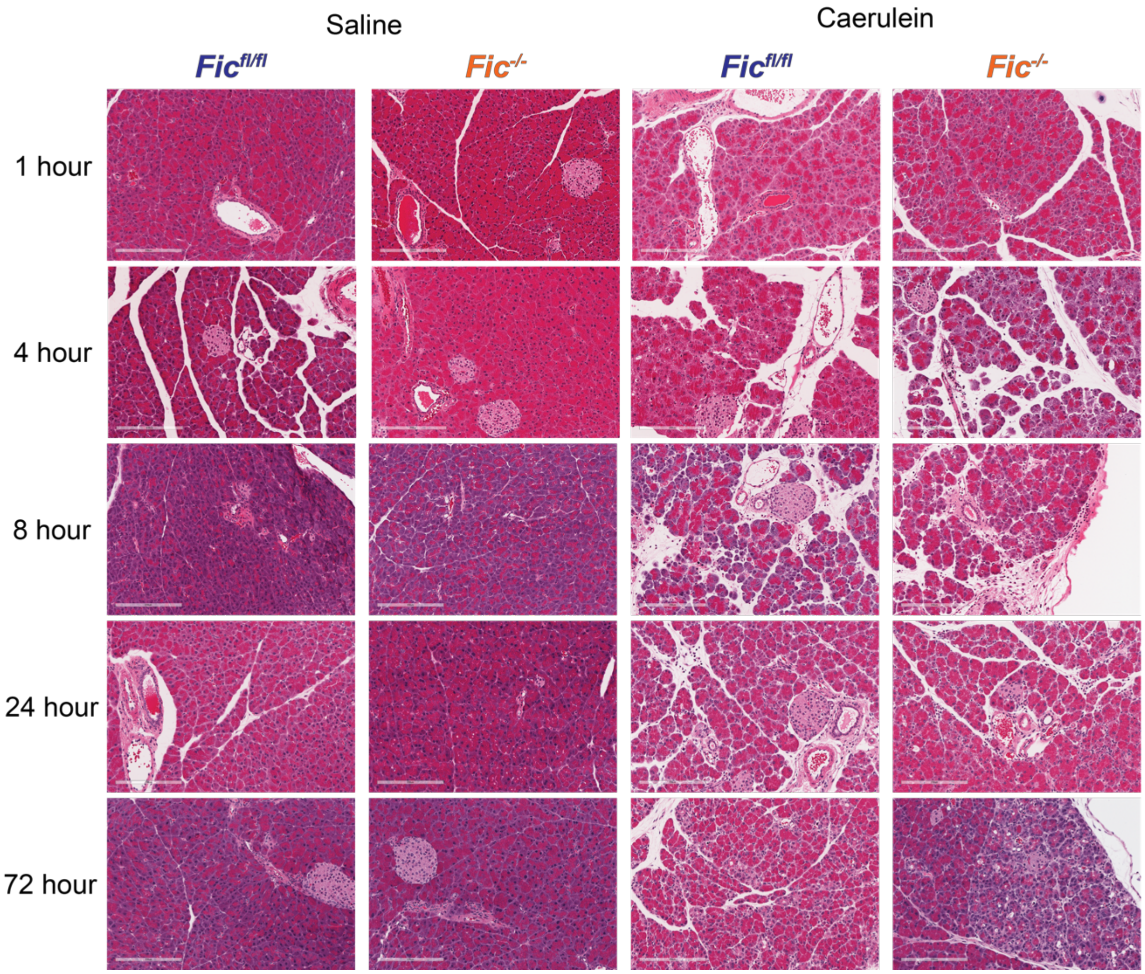

Fig. S13. Representative hematoxylin and eosin stained images of pancreas of *Fic*$^{fl/fl}$ and *Fic*$^{-/-}$ mice treated with saline or caerulein at 1, 4, 8, 24, and 72 hours after first injection. Scale bar, 200μM.

Table S1. Histopathological scoring of caerulein pancreatitis. Average scores+/- S.E.M.

GENOTYPE	TREATMENT	Time	Edema	Inflammation	Necrosis	TOTAL
Fic$^{fl/fl}$	SALINE	1hr	0 ± 0	0 ± 0	0 ± 0	0 ± 0
Fic$^{-/-}$	SALINE	1hr	0 ± 0	0 ± 0	0 ± 0	0 ± 0
Fic$^{fl/fl}$	CAERULEIN	1hr	0 ± 0	0 ± 0	0 ± 0	0 ± 0
Fic$^{-/-}$	CAERULEIN	1hr	0 ± 0	0 ± 0	0 ± 0	0 ± 0
Fic$^{fl/fl}$	SALINE	4hr	0 ± 0	0 ± 0	0 ± 0	0 ± 0
Fic$^{-/-}$	SALINE	4hr	0 ± 0	0 ± 0	0 ± 0	0 ± 0
Fic$^{fl/fl}$	CAERULEIN	4hr	1 ± 0	0 ± 0	0 ± 0	0 ± 0
Fic$^{-/-}$	CAERULEIN	4hr	1 ± 0	0 ± 0	0 ± 0	0 ± 0
Fic$^{fl/fl}$	SALINE	8hr	0 ± 0	0 ± 0	0 ± 0	0 ± 0
Fic$^{-/-}$	SALINE	8hr	0 ± 0	0 ± 0	0 ± 0	0 ± 0
Fic$^{fl/fl}$	CAERULEIN	8hr	1.83 ± 0.40	2.17 ± 0.17	2.5 ± 0.22	6.5 ± 0.72
Fic$^{-/-}$	CAERULEIN	8hr	2.4 ± 0.40	2.2 ± 0.37	2.6 ± 0.40	7.2 ± 1.11
Fic$^{fl/fl}$	SALINE	24hr	0 ± 0	0 ± 0	0 ± 0	0 ± 0
Fic$^{-/-}$	SALINE	24hr	0 ± 0	0 ± 0	0 ± 0	0 ± 0
Fic$^{fl/fl}$	CAERULEIN	24hr	2.25 ± 0.25	2.38 ± 0.18	2.25 ± 0.16	6.88 ± 0.44
Fic$^{-/-}$	CAERULEIN	24hr	2.29 ± 0.18	2.43 ± 0.20	2.14 ± 0.26	6.86 ± 0.51
Fic$^{fl/fl}$	SALINE	72hr	0 ± 0	0 ± 0	0 ± 0	0 ± 0
Fic$^{-/-}$	SALINE	72hr	0.14 ± 0.14	0.29 ± 0.29	0.29 ± 0.28	0.71 ± 0.71
Fic$^{fl/fl}$	CAERULEIN	72hr	0.67 ± 0.21	2 ± 0.26	2 ± 0.26	4.67 ± 0.67
Fic$^{-/-}$	CAERULEIN	72hr	0.71 ± 0.18	1.86 ± 0.34	1.71 ± 0.36	4.29 ± 0.81

Table S2. Primer sequences for the genes analyzed in this study

Gene name	Forward primer sequence	Reverse primer sequence
U36B4	5' cgtcctcgttggagtgaca 3'	5' cggtgcgtcagggattg 3'
Chop	5' ccagaaggaagtgcatcttca 3'	5' actgcacgtggaccaggtt 3'
Atf3	5' tggagatgtcagtcaccaagtct 3'	5' gcagcagcaattttatttctttct 3'
Atf4	5' actctaatccctccatgtgtaaagg 3'	5' caggtaggactctgggctcat 3'
Hmox1	5' caggtgtccagagaaggcttt 3'	5' tcttccagggccgtgtagat 3'
Fgf21	5' cctctaggtttctttgccaacag 3'	5' aagctgcaggcctcaggat 3'
Amy2	5' gcagacctttcattttccaagag 3'	5' cacacggccatttccaaagta 3'
Egr1	5' agcgccttcaatcctcaag 3'	5' tttggctgggataactcgtc 3'
Ask1	5' tgacaccacacaacaaggtct 3'	5' cgaggggtaagcagatggg 3'
Xbp1S	5'ctgagtccgcagcaggt 3'	5' tgtcagagtccatgggaaga 3'
BiP	5' caaggattgaaattgagtccttctt 3'	5' ggtccatgttcagctcttcaaa 3'
Fic	5' gtagacgcactgaatgagttcg 3'	5' tggtgtataagtagtcagcctgg 3'
